# Assessment of skin barrier function using skin images with topological data analysis

**DOI:** 10.1038/s41540-020-00160-8

**Published:** 2020-12-18

**Authors:** Keita Koseki, Hiroshi Kawasaki, Toru Atsugi, Miki Nakanishi, Makoto Mizuno, Eiji Naru, Tamotsu Ebihara, Masayuki Amagai, Eiryo Kawakami

**Affiliations:** 1 Medical Sciences Innovation Hub Program, RIKEN, Yokohama, Kanagawa 230-0045 Japan; 2 Department of Dermatology, Keio University School of Medicine, Shinjuku-ku, 160-0016 Tokyo Japan; 3 School of Medicine, Yokohama City University, Yokohama, 236-0004 Kanagawa Japan; 4 Laboratory for Skin Homeostasis, RIKEN Center for Integrative Medical Sciences, Yokohama, 230-0045 Kanagawa Japan; 5 Dermatology and Cosmeceuticals Sec, KOSÉ Corporation, Kita-ku, 114-0005 Tokyo Japan; 6 Artificial Intelligence Medicine, Graduate School of Medicine, Chiba University, Chiba, 260-8670 Chiba Japan

**Keywords:** Computational science, Diseases, Health care, Applied mathematics

## Abstract

Recent developments of molecular biology have revealed diverse mechanisms of skin diseases, and precision medicine considering these mechanisms requires the frequent objective evaluation of skin phenotypes. Transepidermal water loss (TEWL) is commonly used for evaluating skin barrier function; however, direct measurement of TEWL is time-consuming and is not convenient for daily clinical practice. Here, we propose a new skin barrier assessment method using skin images with topological data analysis (TDA). TDA enabled efficient identification of structural features from a skin image taken by a microscope. These features reflected the regularity of the skin texture. We found a significant correlation between the topological features and TEWL. Moreover, using the features as input, we trained machine-learning models to predict TEWL and obtained good accuracy (*R*^2^ = 0.524). Our results suggest that assessment of skin barrier function by topological image analysis is promising.

## Introduction

The skin provides an effective barrier between the external environment and the body, preventing the entry of pathogens and microorganisms, as well as restricting water loss^1^. Skin barrier research has gained huge momentum after the discovery of the filaggrin mutation (*FLG*) in atopic dermatitis (AD) patients^2^. Filaggrin is an epidermal structural protein critical for skin barrier formation, and the *FLG* mutation is a major risk factor for AD^3^. Studies have identified an association between the *FLG* mutation and asthma and food allergies, such as peanut allergies, even in the absence of AD^3^. Accumulating evidence, including the results of murine studies, suggests that an altered skin barrier is involved in atopic diseases, including AD^4,5^.

Transepidermal water loss (TEWL) is the amount of water that evaporates from the body surface and is most widely used to evaluate skin barrier function^6^. Since healthy skins have the capacity of retaining water, high and low TEWL are indicative of skin barrier dysfunction and intact skin or recovered skin barrier, respectively. Recent studies suggest that TEWL not only reflects the current state of the skin barrier, but it is also a subclinical biomarker for AD and food allergy^7,8^. Since TEWL is sensitive to environmental factors, such as temperature and humidity, examinees are required to wait in the test environment, where temperature and humidity are controlled for a certain period of time (~20 min) before measurement^6^. Therefore, direct measurement of TEWL is not easily implemented as a method to estimate skin barrier function in daily clinical practice, and more practical alternative methods are necessary.

Instead of the direct measurement of TEWL, we considered assessing the skin barrier function by image analysis of the skin surface. Since it is simple to take microscopic pictures of the skin surface, skin barrier assessment by image analysis would be beneficial in daily clinical practice and in subclinical skin care of healthy people. Recently, convolutional neural networks (CNNs) have been reported as very effective in the field of medical image analysis for extracting important features and predicting clinical characteristics^9^. However, CNNs require a vast number of images, as well as significant computational resources and fine-tuning of parameters. In addition, the learning process for interpretation of extracted features is rather difficult^10^. Therefore, we applied topological data analysis (TDA) instead to extract features representing the shape of skin surfaces. TDA is a collection of methods for identifying topological structures in data^11^ and is now considered to be an effective tool to analyze various data in many areas including material science^12^, engineering^13^, and biology^14^. Moreover, TDA has also been applied in medicine to the quantification of tumor shapes^15–17^, finding patterns in genetic data of cancer patients^18^, and characterizing brain artery networks^19^. In dermatology, TDA has been applied to segmenting and classifying skin lesions^20–23^ and quantifying the connectivity of epidermal cells^24^. TDA detects the number of topological features, such as connected components, holes, and cavities, and demonstrates their robustness and magnitude. This information facilitates quantification of the shape and regularity of the skin surface. A study that examined 350 healthy adult women showed that there is a significant difference between populations with high and low TEWL in terms of the number of skin ridges^25^. These findings suggest that structural features in the skin surface contain information associated with skin barrier function and supports our notion that skin barrier function may be assessed from skin images.

In this study, we propose a new skin barrier assessment method using skin images with TDA. We extracted features representing the regularity of skin surface patterns from images of 244 healthy people and predicted their TEWL using machine learning using identified features as predictive variables. We found that the assessment of skin barrier function from skin images using TDA holds promise in improving the accuracy of TEWL prediction.

## Results

### Evaluating skin patterns with TDA

**Fig. 1:**
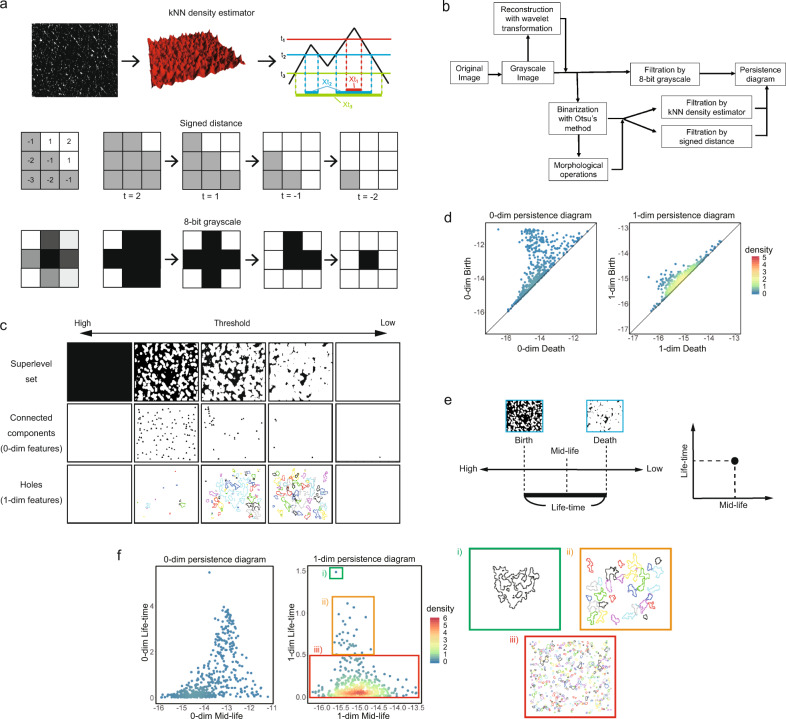
An illustration of feature extraction using TDA. **a** Three filtration functions are illustrated. In the upper row, the density of white pixels is calculated using the kNN density estimator, and elevated regions in the 3D map represent a higher density. As the threshold decreases from *t*_1_ to *t*_3_, the superlevel set (the domain above the threshold) grows from *Xt*_1_ to *Xt*_3_. In the middle and lower rows, the signed distance and the 8-bit grayscale are, respectively, used as filtration functions. As the threshold decreases, the superlevel set (white region) expands. **b** The flowchart of our method. An original image is transformed into a grayscale image. For the kNN density estimator or signed distance as the filtration function, the image is binarized with Otsu’s method. Wavelet transformation and morphological operations can be applied. Following TDA, the persistence diagram is obtained. **c** A schematic diagram of thresholds and superlevel sets. In the upper row, as the threshold decreases, the superlevel set (white region) grows larger. The middle row shows the birthplace of connected components (0-dim topological features), and the lower row shows the holes (1-dim topological features) in the corresponding superlevel set. **d** The information on the detected topological features of each image is summarized in two diagrams called the persistence diagrams. The axes represent the log-scale thresholds where the feature appears (“birth”) and disappears (“death”). The 0-dim persistence diagram represents information of the connected components, and the 1-dim persistence diagram represents that of holes. The color scale represents the density of points in the persistence diagram calculated by the function “kde2d” of the R package MASS. **e** For interpretability and handiness, we transform “birth” and “death” into their mean (mid-life) and their difference (life-time) and plot them. **f** The persistence diagrams with the axes mid-life and life-time. The color scale represents the density of points in the persistence diagram. The three small panels on the right show the holes in each corresponding domain of the 1-dim persistence diagram. The life-time roughly corresponds to the size of holes.

Several TDA algorithms have been proposed in terms of thresholding methods called filtration functions, including the *k*-nearest neighbor (kNN) density estimator, the signed distance, and the 8-bit grayscale value (Fig. 1a). The kNN density estimator calculates the density of white pixels by measuring the distance from the considered pixel to the *k*th nearest white pixel for a fixed integer *k*^26^. The signed distance method assigns the Manhattan distance from the border between black and white areas with a positive sign to a white pixel and a negative sign to a black pixel^27,28^. The 8-bit grayscale value reflects the brightness of a pixel ranging from 0 (black) to 255 (white)^16^. For each threshold value, the superlevel set is defined as the image region where the value of the filtration function is larger than the threshold. TDA analyzes how the shape of the superlevel set changes with a gradually changing threshold.

According to the choice of filtration functions, there are several options for image preprocessing (Fig. 1b). Here, we illustrate the TDA procedure for the case using the kNN density estimator as the filtration function. First, we transformed the images into grayscale and binarized them with Otsu’s method^29^. Wavelet transformation and morphological operations can be applied to remove brightness disproportion and noise.

After preprocessing, we quantified patterns of the images using TDA. There are two kinds of topological features in 2D image analysis: connected components and holes. Connected components (0-dim topological features) are continuously connected regions of white pixels. Holes are continuous loops through a white region surrounding a black region. In our procedure, each filtration function assigns large values to hollow spots, such as sulci cutis, and small values to protuberant spots, such as cristae cutis. Intuitively, holes represent sulci surrounding cristae, and connected components represent connected regions made of sulci. As the threshold decreases, the superlevel set spreads gradually (Fig. 1c, upper row). Connected components appear and merge, and finally, there is only one connected component (Fig. 1c, middle row). Likewise, as the threshold decreases, holes appear, fill up, and finally, there is no hole (Fig. 1c, lower row). We recorded the thresholds in log-scale at the point where the connected components and holes appeared and disappeared, described as *birth* and *death*, respectively.

The popular way to express this extracted information is drawing *persistence diagrams* which show the relationship between birth and death (Fig. 1d)^30^. We plotted the means of birth and death (*mid-life*) and the difference between birth and death (*life-time*) of each topological feature instead of directly plotting birth and death (Fig. 1e, f) since they were easy to interpret. The mid-life indicates the threshold at which the feature exists. Features with a large life-time can be regarded as important structures because it is likely that these are not noise^31^. Also, the life-time of holes roughly correlates with the size of corresponding holes (Fig. 1f, right three panels). On the other hand, the distribution of mid-life is related to the regularity of the image. Since features visible in the original image appear at the early stage with high mid-life, if the image has a regular ring-shaped structure, many holes appear convergently at the range of high mid-life.

### Relationships between TEWL and persistence diagrams

**Fig. 2:**
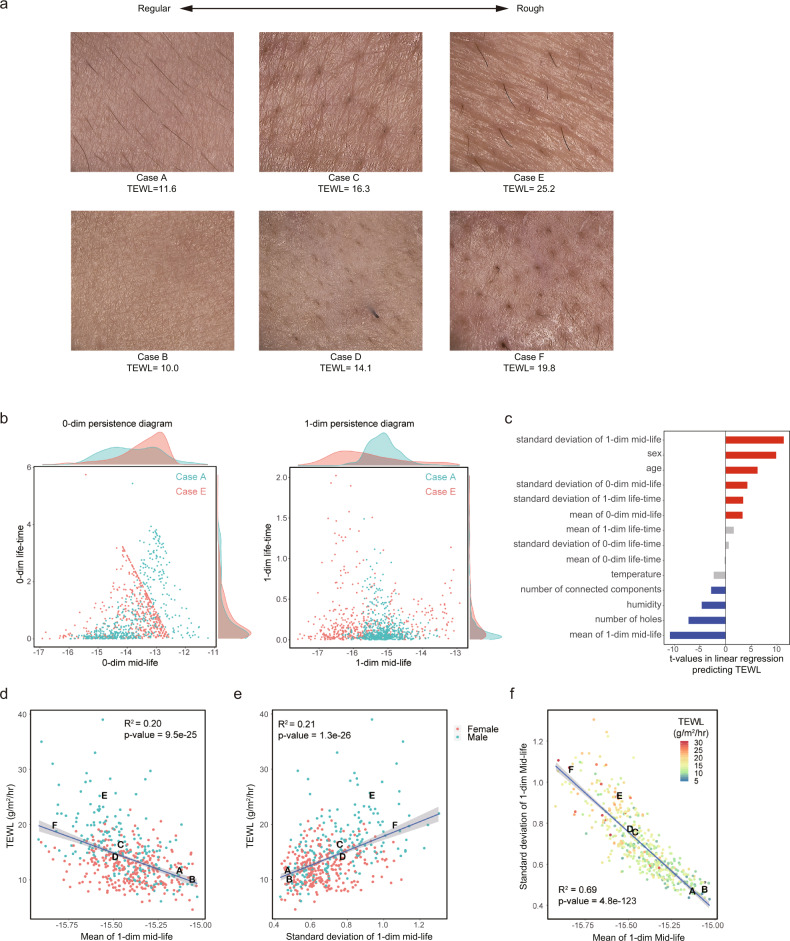
Relationships between TEWL and features of skin images extracted using the kNN density estimator. **a** Typical examples of skin images. Cases A and B show relatively regular skin texture and low TEWL, and cases E and F show relatively rough skin surfaces and high TEWL. The written consent was obtained for publication of the photographs. **b** The persistence diagrams of cases A and E with marginal distributions. It is remarkable that the mid-life of case A has a sharper peak with a higher value range than that of case E. **c** The *t*-value of each variable is calculated using simple linear regression predicting TEWL. As explanatory variables, we used the mean and standard deviation of mid-life and life-time for 0-dim and 1-dim topological features, the number of all connected components and holes, age, and sex. Variables with a false discovery rate (FDR) larger than 0.01 are colored red (if its *t*-value is positive) or blue (if its *t*-value is negative). **d**, **e** The results of simple linear regression predicting TEWL from the mean and the standard deviation of 1-dim mid-life with the coefficient of determination (*R*^2^) and *p*-value. Since sex is the most important variable in determining TEWL other than the topological features of the images, each point is colored red or blue according to the subject’s sex. The points representing cases A–F are also labeled. The shaded areas represent the 95% confidence intervals of the regression. **f** The mean and the standard deviation of 1-dim mid-life are linearly related, and a clear observed TEWL trend change can be seen along the regression line. The shaded area represents the 95% confidence interval of the regression.

We compared the distributions of mid-life and life-time on 0-dim and 1-dim topological features. For illustration, we show six typical samples of cheek skin images (Fig. 2a). We found clear differences in their persistence diagrams. In particular, the distribution of 1-dim mid-life for case A had a sharper peak in the higher value range than that for case E (Fig. 2b). Because case A had a regular texture, holes appeared intensively at the range of high mid-life. On the other hand, case E showed a wavy pattern and very little texture, and holes appeared loosely at the range of low mid-life.

To investigate the correlations between TEWL and topological features, we performed linear regression analysis. Although linear regression only captures linear relationships between variables, it enables us to intuitively understand whether two variables have a significant relationship, how closely they are correlated, and whether the correlation is positive or negative^32^. As the comparison of case A and case E suggested, there was a strong positive correlation between TEWL and the standard deviation of 1-dim mid-life (Fig. 2c, d) and strong negative correlation between TEWL and the mean of 1-dim mid-life (Fig. 2c, e). These correlations suggest that TDA can detect regularity of skin texture patterns which are associated with skin barrier integrity. Interestingly, the correlations between TEWL and some topological features were stronger than those of background factors, such as age and sex, and environmental factors, such as temperature and humidity. This indicates that skin images include substantial information associated with skin barrier function. The mean and standard deviation had an inverse linear relationship, and a clear trend for TEWL changes could be seen along the regression line (Fig. 2f), suggesting that this line may be considered an indicator of the regularity of skin surfaces. From these results, we concluded that the topological features extracted from skin images using TDA provide essential information associated with skin barrier function.

We also analyzed the relationships between the moisture content of the stratum corneum, and the features extracted from skin images (Supplementary Fig. 1a, b). The moisture content was measured using two devices, namely, the Corneometer (Model CM825; Courage & Khazaka Electronic, Cologne, Germany) and the Skicon (Model 200EX-USB; YAYOI, Tokyo, Japan). We compared these two devices owing to differences in their mechanisms. The Corneometer uses electrical capacitance, whereas the Skicon uses high-frequency conductance to assess the level of hydration^33,34^. Reports suggest that the Skicon is more sensitive to hydration dynamics compared with the Corneometer; however, the latter is useful for the measurement of very dry skins^35^. In contrast to TEWL, the moisture content was not strongly related to skin images with both devices. Instead, the moisture content was more associated with the environmental condition.

### Prediction of TEWL with machine learning

**Fig. 3:**
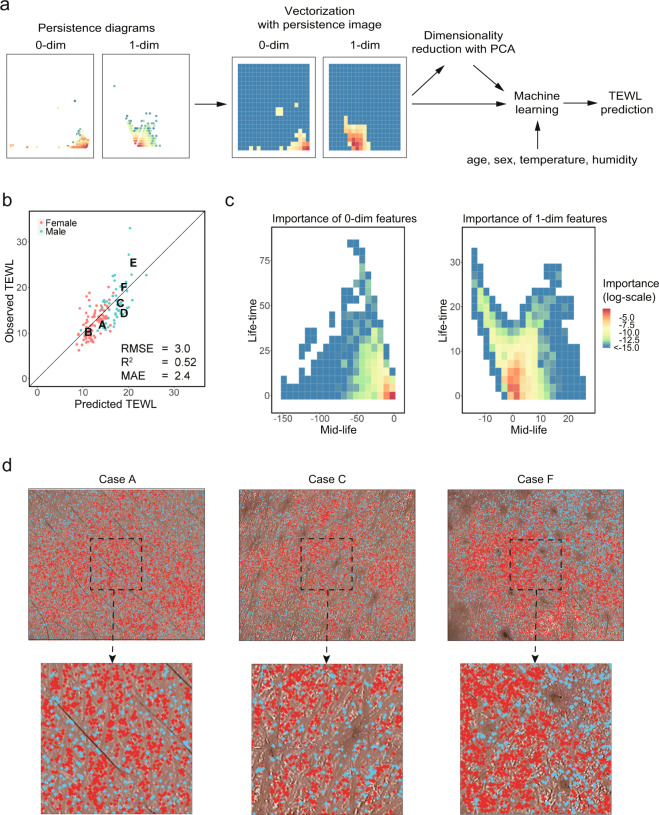
Prediction of TEWL. **a** The protocol for TEWL prediction using machine learning. Each persistence diagram was vectorized by the persistence image. Then, when using principal component analysis (PCA), each vector was reduced to a low-dimensional vector with PCA and combined with age, sex, temperature, and humidity to create a feature vector. Using the feature vectors, several machine-learning analyses were performed to predict TEWL. **b** Predicted vs. observed plots of TEWL produced by random forest regression. The values of cases A–F in Fig. 2a are also labeled. As indicators of accuracy, the coefficient of determination (*R*^2^), the root mean squared error (RMSE), and the mean absolute error (MAE) are shown. **c** The variable importance of each region in the persistence diagrams is calculated by the random forest regression model according to how much the variable contributed to the regression of TEWL. **d** For cases A, C, and F in Fig. 2a, the birth position of connected components (red) and the death position of holes (blue) in the most important regions of the persistence diagrams are drawn (upper row) and enlarged (lower row).

In the previous section, we showed that topological features on skin images have a significant correlation with TEWL. Given this, we used the features in the persistence diagrams to predict TEWL with machine-learning algorithms. The process of prediction is shown in Fig. 3a. First, for cross-validation, we partitioned all images into 70% training data (997 images of 170 people) and 30% test data (431 images of 74 people). We investigated the relationships between TEWL and summarized values (means and standard deviations of the mid-life and life-time) of persistence diagrams; however, these values omit a significant amount of information that may have important relationships with skin conditions. Since we cannot apply machine-learning algorithms directly to persistence diagrams due to the space of persistence diagrams lacking the vector space structure (e.g., each persistence diagram has a different number of points), we had to vectorize the persistence diagrams before applying machine-learning algorithms.

Various vectorization methods for persistence diagrams have been proposed. For example, the persistence landscape embeds persistence diagrams into a Banach space made of piecewise-linear functions^36^; kernel methods were used to apply kernel-based machine-learning methods and statistical concepts to persistence diagrams^37,38^; functions defined using tropical geometry were also utilized to represent persistence diagrams without loss of information^39^. Among many vectorization methods, we applied the *persistence image* to vectorize persistence diagrams^40^ because the persistence image embeds each persistence diagram into a finite-dimensional Euclidean space that is easy to handle. Besides, another approach exists that directly combines TDA and neural networks^41^. The combination of the persistence image and machine learning enabled us to easily analyze which regions of persistence diagrams were important in the prediction and where important features (connected components and holes) resided in the original images^27^.

After vectorization of the persistence diagrams, we combined each generated vector with age, sex, temperature, and humidity to make the feature vector for each subject and applied several machine-learning methods to construct TEWL prediction models. Since high-dimensional data that is composed of highly correlated variables often causes inefficient prediction due to multicollinearity and overfitting, we applied a principal component analysis (PCA) to the features extracted from images and extracted the most important components whose proportions of variance were larger than 0.01. Among the several machine-learning algorithms and linear regression model, the random forest regression model with PCA was the best prediction model according to the coefficient of determination (*R*^2^) (Supplementary Table 1). Furthermore, the choices of several filtration functions, preprocessing methods, and vectorization methods have been considered and summarized in Supplementary Tables 2 and 3. The preprocessing procedure with the best performance was (1) signed distance as the filtration function, (2) persistence image with a standard deviation of 0.1 as the vectorization method, and (3) without wavelet transformation or morphological operations in the preprocessing. With this procedure, the random forest regression model predicted TEWL of the test data with high accuracy (*R*^2^ = 0.524; Fig. 3b). Combining the three feature vectors obtained by the three filtration functions (each under the optimal combination of other methods) did not improve the prediction accuracy (*R*^2^ = 0.512). This is likely because all three filtration functions extract quite similar features of the skin image and, therefore, the information obtained using each method overlaps.

The variable importance of each region in the persistence diagrams was calculated using the random forest regression model (Fig. 3c). The regions that contributed significantly to the regression of TEWL had high importance. For three subjects, we extracted only those features in the regions of highest importance and drew their locations over the original images (Fig. 3d). The locations of connected components were represented by their birth positions (i.e., the locations where they appear), and those of holes were represented by their death positions (i.e., the locations where they are filled in)^27^. We confirmed that large structures such as pores and hairs were not included in the important features.

We also predicted the moisture content of the stratum corneum with the random forest regression model from the same variables as for TEWL (Supplementary Fig. 1c, d). The choices of several filtration functions, preprocessing methods, and vectorization methods were considered and are summarized in Supplementary Tables 4 and 5. Although the accuracy was lower than the prediction of TEWL, the moisture content can also be predicted with *R*^2^ = 0.219 for the Corneometer and *R*^2^ = 0.364 for the Skicon. This is consistent with the linear regression results, where the moisture content measured with the Skicon had a stronger association with the topological features and also with the environmental condition.

## Discussion

In this study, we predicted TEWL from skin images with good accuracy by combining TDA and machine learning. An advantage of this method is that it takes far less time than the direct measurement of TEWL. In contrast to the direct measurement of TEWL, which requires subjects to wait in the test environment with controlled temperature and humidity for about 20 min, our method requires only a short time for taking a picture of the subject and a few seconds for analyzing the image. Therefore, we believe that our method can be applied in daily clinical practice and to the skin care of healthy people. Another advantage of our method is that it requires a relatively small number of images. In this study, we used only 997 images of 170 subjects to train the prediction model. Typically studies using CNNs in dermatology use 10,000–100,000 skin images to train models^42–44^, which is sometimes problematic in clinical image analysis where not so many images of the same standard are available for training. TDA extracts predefined essential information on the skin surface structures and does not require learning for feature extraction. Because of this characteristic, our method based on TDA can be easily transferred to new projects which utilize images of different standards. Feature extraction methods such as TDA are suited for situations where a small number of images is available and clear features exist; conversely, deep learning is suited for situations where many images are available and defining specific features is difficult. Therefore, it is essential to use deep-learning and non-learning feature extraction methods in parallel when dealing with medical images of various types and sizes.

Our results showed that TDA can quantify the regularity of skin surfaces, which correlates with TEWL. A previous study also applied TDA to detect the patterns of the microsurface structure of the gastrointestinal tract; images were classified according to their patterns into three groups with variable risk for cancer (oval, tubular, and irregular patterns with no, low, and high risk, respectively). Approximately 90% of the classification matches were performed by medical doctors^15^. Moreover, in cardiac image analysis, a study applying TDA to computed tomography images successfully extracted the shape of the trabeculae, the fine muscle columns on the ventricular walls which had been missed by previous methods^45^. These results and ours suggest that TDA is suitable for the image analysis of organs with fine structures. Since there are many organs with fine structures in the body, such as the lung, liver, and brain, there are many potential applications of TDA in the field of medicine. In dermatology, several studies applied TDA to the malignancy classification of melanomas using skin images taken by dermatoscopes or stereomicroscopes^20–22^. Another study proposed a method of applying TDA to classify seven skin diseases including melanomas and basal cell carcinomas^23^. These studies have shown that skin image analysis using TDA is useful for the qualitative assessment of skin diseases. Our application of TDA, on the other hand, allowed us to quantitatively evaluate the skin phenotype. This will lead not only to the stratification of existing skin diseases but also to the prediction of pathological changes in chronic skin diseases such as AD. Quantification of skin structures using TDA may lead to the establishment of objective diagnostic criteria for skin diseases. In this study, the moisture content of the stratum corneum did not correlate strongly with topological features of the skin. This is probably because the moisture content reflects the state of the deeper layer of the stratum corneum, which does not appear in the surface^46^. The Raman microspectrometer (Model 3510; River Diagnostics BV, Rotterdam, The Netherlands) has been used to determine molecular concentration profiles in the deep skin^47,48^. Combining topological features of the skin surface with such deep skin information may allow for more detailed skin condition monitoring and stratification.

Historically, dermatology has evolved through the observation of body surfaces by specialists^49^. However, recent developments in molecular biology and genomic sciences have revealed mechanisms and causative genes of skin diseases, and many biologics have appeared^49^. For precision medicine considering these mechanisms, it is necessary to objectively evaluate the skin condition and systematically select treatment that is suitable for each individual patient. Although the present study is exclusively designed for healthy people, the methodology may be extrapolated to dermatological disease research for precision medicine. More samples may be needed to reflect the diversity of the patients; in addition to phenotypes, genetic factors such as *FLG* mutations should also be analyzed to improve understanding of the disease pathology. Deep clinical phenotyping based on TDA can provide a basis for precision medicine in dermatology by quantifying clinical characteristics and associating them with molecular biological knowledge.

## Methods

### Subjects and measurement

We recruited 244 healthy subjects between the ages of 0 and 64 years. Among them, 143 were women, and 101 were men; 132 were from Akita, and 112 were from Tokyo, Japan (Supplementary Table 6). We only chose subjects who did not use external topical medicines and did not have any skin diseases or other factors such as warts and stains in the measured region. For each subject, measurements were performed twice, in June and December 2018. We obtained informed consent for analysis and publication of measured data and skin photographs from all participants and ethical approval from the Kenshokai ethical committee for data acquisition [IRB no. 20180810-2 and H30-044] and from Keio University School of Medicine Ethics Committee for data analysis [IRB no. 20160191-6] in accordance with the Declaration of Helsinki. The procedure of the measurement performed in June and December involved certain steps. Before measurement, subjects washed their face using makeup remover and facial wash. For infants who could not wash their faces themselves, we wiped their faces with damp cotton wool twice. Subjects waited for more than 15 min in the test room with temperature and humidity kept to 20.0 °C and 50%, respectively. We took three pictures of the left cheek (the intersection point of the horizontal line through the inferior margin of the nose and the vertical line through the left edge of the left eye) using a microscope and measured TEWL and the moisture content of the stratum corneum of the same region. We used a digital microscope (Model KH-8700; Hirox, Tokyo, Japan) and a vapometer (Model SWL5001JT; Delfin, Technologies Ltd, Kuopio, Finland) to measure TEWL and the Corneometer (Model CM825; Courage & Khazaka Electronic, Cologne, Germany) and Skicon (Model 200EX-USB; YAYOI, Tokyo, Japan) to measure the moisture content of the stratum corneum. We measured TEWL three times and the moisture content of the stratum corneum five times per subject and used their medians in the subsequent analysis.

### Image processing

We processed skin images using the Python packages OpenCV^50^ and PyWavelets^51^. We trimmed the images into 1400 × 1200 pixels to delete scale bars and transformed them into grayscale using the OpenCV function “cv2.cvtColor” and “cv2.COLOR_BGR2GRAY.” In the wavelet transformation, the grayscale image was decomposed into levels from 0 (coarsest) to 10 (finest). Wavelet coefficients at coarse resolutions represent large structures of the image, including disproportionate light intensity. The image was then reconstructed using only some of these levels (Supplementary Fig. 2).

The images were binarized using Otsu’s method. In the morphological operations, the eroding operation was applied using the OpenCV function “cv2.erode” to expand the black region with the structuring element obtained by the OpenCV function “cv2.getStructuringElement(cv2.MORPH_CROSS,(3,3)).”

### Application of TDA using the kNN density estimator

The kNN density estimator was applied as the filtration function using the R package TDA^52^. The specific process was as follows: We set a grid spacing of 10 pixels on an image. On each grid point, the density of white pixels was estimated by measuring the *k*th nearest white pixel with the parameter *k* set to 100. We gradually decreased the threshold from ∞ to −∞ and recorded the thresholds in log-scale at the point where the connected components and holes appeared and disappeared as birth and death. We calculated the mean (mid-life) and the difference (life-time) of these and plotted them to draw the persistence diagrams. We calculated the means and standard deviations of the mid-life and life-time, respectively.

### Linear regression analysis

After the skin images had been processed by grayscale transformation, wavelet transformation using levels 4–10, Otsu’s method, eroding operation for five times, and TDA with the kNN density estimator, we performed 14 separate simple linear regression analyses using the R lm function to predict TEWL from 14 explanatory variables, such as sex, age, temperature, humidity, the number of all connected components and holes, and the means and standard deviations of mid-life and life-time of connected components and holes. To investigate the influences of environmental factors on TEWL, we included the temperature and humidity as explanatory variables; these were the averages of the daily temperature and humidity over one month. We calculated the *t*-value and the two-sided *p*-value of each regression. The false discovery rate was calculated using the R p.adjust function with the method of Benjamini and Hochberg^53^.

### Comparison of machine learning models for predicting TEWL

First, we performed machine learning using the R package caret to investigate which algorithm performs best^54^. We removed the meaningless dimensions with very low variances from the count data of the partitioned persistence diagrams using the caret nearZeroVar function. We performed PCA using the R prcomp function. The eight most important components, namely, PC1–PC8, with contribution ratios larger than 0.01 were used. Then, we created two feature vectors for each sample. One was made of the count data together with age, sex, temperature, and humidity, and the other was made of PC1–PC8 together with age, sex, temperature, and humidity. We used each feature vector, respectively, to predict TEWL and compared their accuracies. We split all data into 70% training data and 30% test data using the R sample function to evaluate the accuracy of prediction. We constructed several models for predicting TEWL using the caret methods “rf” (random forest), “svmRadial” (support vector machine with Gaussian kernel), “enet” (elastic net), “xgbLinear” (gradient boosting using linear functions), “xgbTree” (gradient boosting using tree models), “nnet” (neural network), and “lm” (linear model). Parameter tuning of each model was performed using 10 separate 10-fold cross-validations. The best parameters were chosen according to the root mean square error (RMSE). Each trained model predicted TEWL from each image. Because we took three pictures of each subject in each measurement, we chose the median of the three predicted values as the genuine predicted TEWL of the subject. We evaluated the prediction models by calculating RMSE, the coefficient of determination (*R*^2^), and the mean absolute error (MAE). Since the random forest performed best, we used it as the prediction algorithm of TEWL in the following procedures. To apply the random forest afterwards, we used the RandomForestRegressor function of the Python package scikit-learn to predict TEWL because it is easier to speed up by parallelization than caret^55^.

### Comparison of vectorization methods, filtration functions, and preprocessing methods

Next, two vectorization methods of persistence diagrams were considered: counting points in each region and persistence image. The dynamic range of persistence diagrams was partitioned into 20 × 20 regions. In the persistence image, for each point in the persistence diagram, we associated a Gaussian distribution centered at the point with the standard deviation set to 0.1 or 1. Then, the distribution was multiplied by linear weighting function which is 0 at the *x*-axis (i.e., where life-time equals 0) and 1 at the maximum life-time of all persistence diagrams. Finally, all the weighted distributions for all points in the persistence diagram were added and integrated over each region to obtain a 400-dimensional vector. We implemented the persistence image by modifying the Python package persim of scikit-tda^56^.

Furthermore, the choice of algorithms of TDA was considered. In addition to the kNN density estimator, the signed distance and 8-bit grayscale were considered as the filtration functions. The signed distance and 8-bit grayscale were applied using the Python package HomCloud (http://www.wpi-aimr.tohoku.ac.jp/hiraoka_labo/homcloud-english.html).

Finally, the choice of preprocessing methods was considered. We assessed the performance of prediction with or without the wavelet transformation and morphological operations. Additionally, to investigate the effect of parameters in the wavelet reconstruction, seven combinations of levels were examined.

### Calculation of the variable importance

The variable importance was calculated using the random forest regression model, which had the vectorized data of persistence diagrams, age, sex, temperature, and humidity as explanatory variables. We obtained an average of the variable importance calculated 10 times with different test data selected randomly. When using PCA, the importance of the regions of persistence diagrams was calculated from the importance of principal components. The information of birth positions and death positions was obtained using the HomCloud function “homcloud.interface.draw_birthdeath_pixels_2d.”

## Supplementary information

Supplemental Material

nr-reporting-summary

## Data Availability

Except for skin images, all data used in this study are included in the GitHub repository (https://github.com/kosekei/skin_TDA). The skin images used in this study are available from the authors upon request.

## Code availability

All scripts used in this study are included in the GitHub repository (https://github.com/kosekei/skin_TDA).
