# Supplementary material for: Assessment of skin barrier function using skin images with topological data analysis
Source: NPJ Syst Biol Appl. 2020 Dec 18;6:40. doi: 10.1038/s41540-020-00160-8 (PMC7749164; doi:10.1038/s41540-020-00160-8)
Supplement: Supplementary file 1 — Supplemental Material [file 41540_2020_160_MOESM1_ESM.pdf]

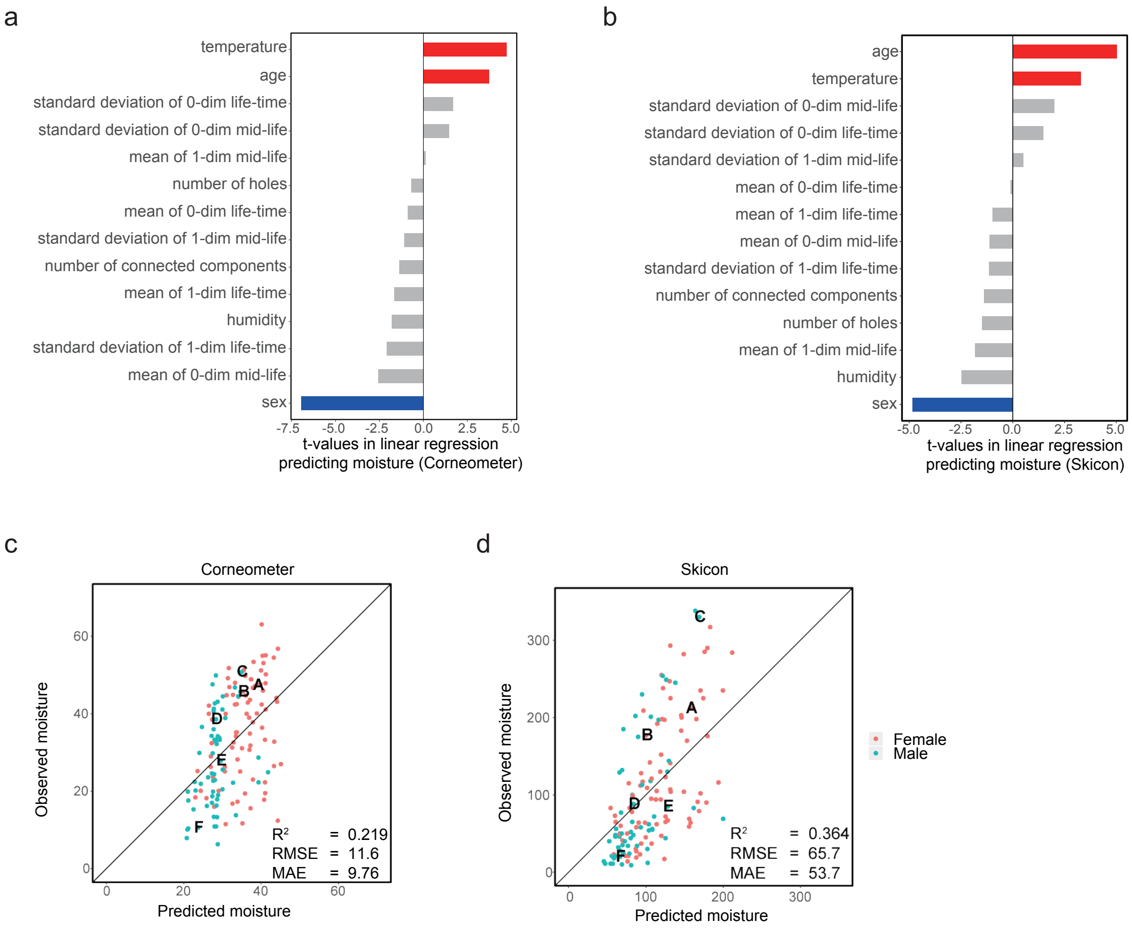

Supplementary Figure 1. Relationships between moisture content of the stratum corneum and extracted features of skin images, age, sex, temperature, and humidity. **a, b**. t-value of each variable calculated by simple linear regression predicting the moisture content measured using the Corneometer (a) and Skicon (b). As explanatory variables, we used the mean and standard deviation of mid-life and life-time of 0-dim and 1-dim topological features, the number of all connected components, the number of holes, age, sex, temperature, and humidity. Variables with a false discovery rate (FDR) larger than 0.01 are colored red (if its t-value is positive) or blue (if its t-value is negative). **c, d.** Predicted vs. observed plots of moisture content measured using the Corneometer (c) and Skicon (d). Moisture content of test data was predicted using random forest regression from the extracted features of skin images, age, sex, temperature, and humidity. The points representing cases A-F in Fig. 2a are labelled. As indicators of accuracy, the coefficient of determination (R^2), the root mean squared error (RMSE), and the mean absolute error (MAE) are shown.

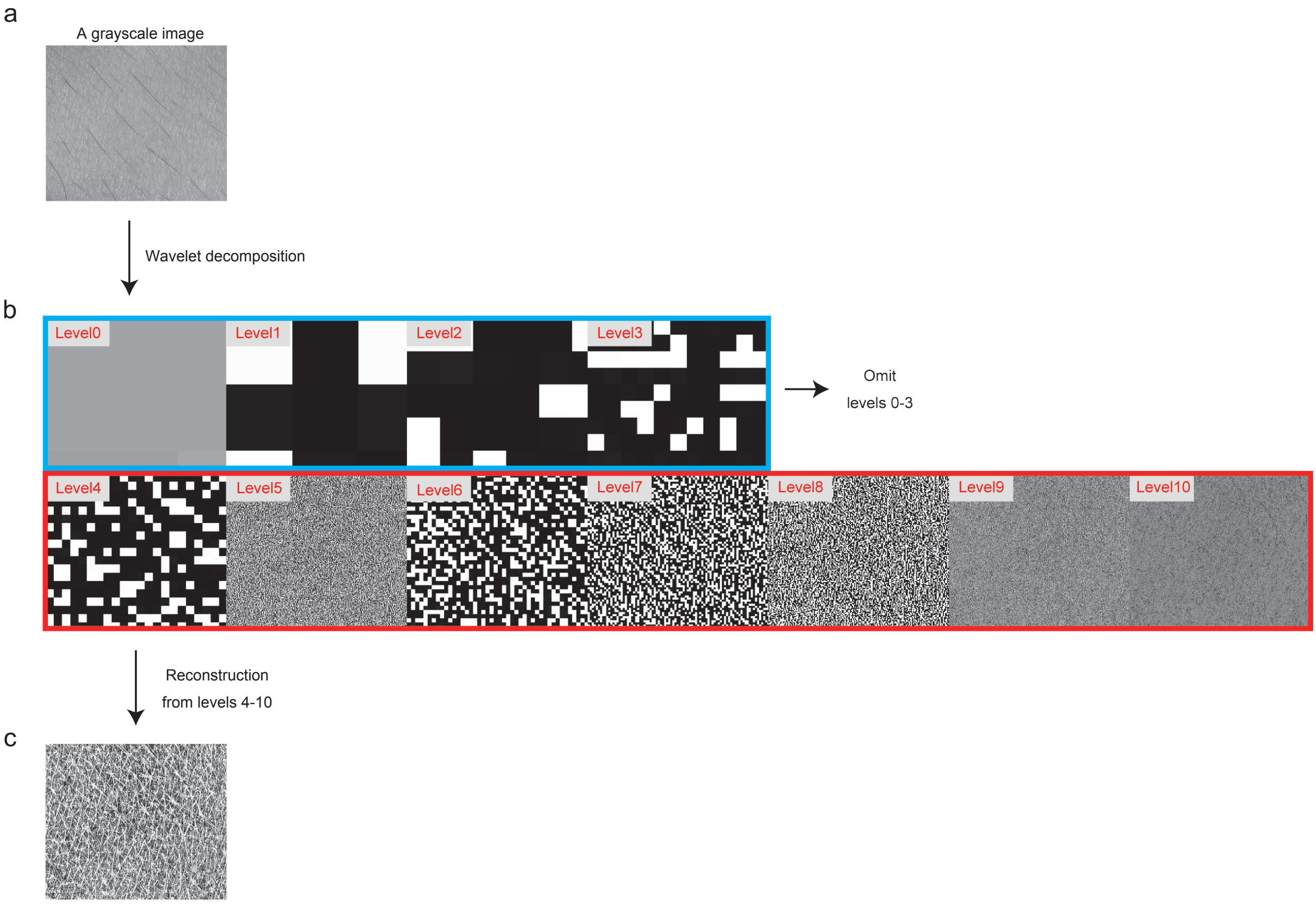

Supplementary Figure 2. Illustration of the wavelet transformation. **a**. A skin image converted into grayscale was used as the input. **b**. The image was decomposed into levels from 0 (coarsest) to 10 (finest), and some of them were removed (e.g., levels 0-3 were omitted in this illustration). **c.** The image was reconstructed from the remaining wavelet coefficients.

Supplementary Table 1. The accuracies of TEWL prediction with several machine learning methods with or without dimensionality reduction by PCA.

Machine Learning Method	Dimensionality reduction	RMSE	R^2	MAE
Random Forest	without PCA	**3.40**	0.424	**2.55**
	with PCA	3.53	**0.444**	2.62
Support vector machine	without PCA	3.74	0.266	2.82
	with PCA	3.92	0.409	2.83
Elastic net	without PCA	3.59	0.330	2.77
	with PCA	3.53	0.354	2.77
Neural network	without PCA	4.20	0.207	3.26
	with PCA	4.28	0.186	3.44
Boosting (tree)	without PCA	3.51	0.408	2.65
	with PCA	3.46	0.376	2.68
Boosting (linear)	without PCA	3.59	0.393	2.71
	with PCA	3.53	0.354	2.77
Linear model (base line)	without PCA	4.66	0.203	3.63
	with PCA	3.51	0.381	2.77

RMSE, Root mean squared error; R^2, Coefficient of determination; MAE, Mean absolute error

Supplementary Table 2. The accuracies of TEWL prediction with several combinations of filtration functions, preprocessing methods and vectorization methods. In each case, we reduced dimensionality of vectors using PCA after vectorization and then applied random forest regression to predict TEWL.

Filtration function	Wavelet transformation	Morphological operations	Prediction accuracy (R^2)		
			Count data	Persistence image	
				SD = 1	SD = 0.1
kNN density estimator (R package TDA)	Yes	Yes	0.449	0.407	0.412
	Yes	No	0.439	0.478	0.472
	No	Yes	0.450	0.497	0.449
	No	No	0.450	0.476	0.437
Signed distance (homcloud)	Yes	Yes	0.445	0.432	0.439
	Yes	No	0.459	**0.513**	0.464
	No	Yes	**0.468**	0.483	0.485
	No	No	0.437	0.420	**0.524**
8-bit grayscale (homcloud)	Yes	Not applicable	0.449	0.506	0.501
	No		0.449	0.447	0.462

R^2, Coefficient of determination; SD, standard deviation of normal distributions used in persistence image

Supplementary Table 3. The accuracies of TEWL prediction using different levels of wavelet decomposition. We decomposed each image from level 0 (coarsest) to 10 (finest) and then reconstruct the image with some of the levels. We applied TDA using signed distance (homcloud) without morphological operations. We vectorized persistence diagrams by persistence image (SD = 1).

Decomposition levels used to reconstruct images		Prediction Accuracy (R^2)
minimum	maximum	
0	10	0.420
2	10	0.512
4	10	**0.513**
6	10	0.471
0	8	0.478
0	8	0.452
4	8	0.472

Supplementary Table 4. The accuracies of prediction of moisture content measured using the Corneometer with several combinations of filtration functions, preprocessing methods and vectorization methods. In each case, we reduced dimensionality of vectors using PCA after vectorization and then applied random forest regression to predict moisture content.

Filtration function	Wavelet transformation	Morphological operations	Prediction accuracy (R^2)		
			Count data	Persistence image	
				SD = 1	SD = 0.1
kNN density estimator (R package TDA)	Yes	Yes	0.173	0.214	0.186
	Yes	No	0.192	0.182	**0.205**
	No	Yes	**0.196**	0.186	0.184
	No	No	0.188	0.218	0.196
Signed distance (homcloud)	Yes	Yes	0.187	**0.219**	0.180
	Yes	No	0.195	0.169	0.171
	No	Yes	0.190	0.183	0.187
	No	No	0.189	0.211	0.204
8-bit grayscale (homcloud)	Yes	Not applicable	0.154	0.156	0.199
	No		0.162	0.188	0.199

R^2, Coefficient of determination; SD, standard deviation of normal distributions used in persistence image

Supplementary Table 5. The accuracies of prediction of moisture content measured using the Skicon with several combinations of filtration functions, preprocessing methods and vectorization methods. In each case, we reduced dimensionality of vectors using PCA after vectorization and then applied random forest regression to predict moisture content.

Filtration function	Wavelet transformation	Morphological operations	Prediction accuracy (R^2)		
			Count data	Persistence image	
				SD = 1	SD = 0.1
kNN density estimator (R package TDA)	Yes	Yes	0.276	0.274	0.286
	Yes	No	**0.331**	0.354	0.320
	No	Yes	0.302	0.316	0.310
	No	No	0.296	0.328	**0.341**
Signed distance (homcloud)	Yes	Yes	0.269	0.278	0.293
	Yes	No	0.325	0.306	0.314
	No	Yes	0.298	0.298	0.283
	No	No	0.315	0.347	0.339
8-bit grayscale (homcloud)	Yes	Not applicable	0.298	0.304	0.301
	No		0.326	**0.364**	0.342

R^2, Coefficient of determination; SD, standard deviation of normal distributions used in persistence image

Supplementary Table 6. The distribution of age, sex and measurement locations of subjects.

Age		0-9	10-19	20-29	30-39	40-49	50-59	60-64	Total
Tokyo	Female	13	12	8	7	24	4	0	68
	Male	10	10	1	7	8	7	1	44
Akita	Female	14	14	17	14	8	5	3	75
	Male	13	11	3	8	12	7	3	57
Total		50	47	29	36	52	23	7	244
